# Loganin alleviates testicular damage and germ cell apoptosis induced by AGEs upon diabetes mellitus by suppressing the RAGE/p38MAPK/NF‐κB pathway

**DOI:** 10.1111/jcmm.15198

**Published:** 2020-04-19

**Authors:** Yuping Chen, Ni Jiao, Ming Jiang, Liping Liu, Yihui Zhu, Hongyan Wu, Jing Chen, Yingxue Fu, Qiu Du, Huiqin Xu, Jihu Sun

**Affiliations:** ^1^ Department of Basic Medical Science Jiangsu Vocational College of Medicine Yancheng China; ^2^ Chemistry and Life Science College Nanjing University Jinling College Nanjing China; ^3^ College of Pharmacy Nanjing University of Chinese Medicine Nanjing China; ^4^ Affiliated Hospital of Nanjing University of Chinese Medicine Nanjing China

**Keywords:** advanced glycation end products, apoptosis, diabetes, loganin, receptor for AGEs, testicular damage

## Abstract

Diabetes mellitus (DM) damages male reproduction at multiple levels, such as endocrine secretion, spermatogenesis and penile erection. We herein investigated the protective effects and mechanism of loganin targeting the advanced glycation end products (AGEs)/receptor for AGEs (RAGE)/p38 mitogen‐activated protein kinase (p38MAPK)/NF‐κB signalling pathway. Loganin relieved the general DM symptoms and decreased the blood glucose level of KK‐Ay DM mice. Haematoxylin‐eosin staining demonstrated that loganin ameliorated testicular histology and function and enhanced the activities of testis‐specific markers lactate dehydrogenase (LDH), acid phosphatase (ACP) and gamma‐glutamyl transferase (γ‐GT). Loganin also showed evident anti‐oxidative stress, anti‐apoptotic and anti‐inflammatory effects on DM‐induced reproductive damage by restoring glutathione (GSH) level and superoxide dismutase (SOD) activity, as well as reducing reactive oxygen species (ROS) level and Bax/Bcl‐2 ratio in vivo and in vitro. Western blotting exhibited that loganin significantly inhibited the AGEs/RAGE/p38MAPK/NF‐κB signalling pathway. Acridine orange and ethidium bromide staining (AOEB) and Western blotting showed that loganin in combination with inhibitors of RAGE, p38MAPK and NF‐κB exerted stronger anti‐apoptotic effects on AGE‐induced GC‐2 cell damage compared with loganin alone. In conclusion, loganin can protect against DM‐induced reproductive damage, probably by suppressing the AGEs/RAGE/p38MAPK/NF‐κB pathway.

## 1 INTRODUCTION

Diabetes mellitus (DM) is a prominent metabolic disorder affecting 425 million people worldwide in 2017, a number expected to rise to 629 million by 2045.^1^ Although the effects of DM on the reproductive system have long been controversial, it is now accepted that long‐term uncontrolled DM with sustained high blood glucose level can cause testicular damage by inducing various micromolecular changes such as erectile dysfunction, retrograde ejaculation, loss of libido and abnormal sperm production that may lead to male infertility.^2^, ^3^, ^4^ A retrospective study reported subfertility in 51% of male diabetics.^5^ Another study showed that diabetic males had significantly higher rates of primary (16%) and secondary (19.1%) infertility than those of subjects without DM.^6^ Although DM is an important cause for male infertility, the underlying mechanism is still unclear. The apoptosis of germ cells and oxidative stress are two major events involved in DM‐induced testicular damage. In DM rats, lipid peroxidation and reactive oxygen species (ROS) overproduction are promoted, and the activities of superoxide dismutase (SOD), catalase and glutathione (GSH) are inhibited.^7^, ^8^ Besides, Chen et al reported that the apoptotic rate of germ cells and Bax/Bcl‐2 ratio in DM rats exceeded those of normal controls.^9^

Advanced glycation end products (AGEs) produced by non‐enzymatic reactions between the sugar and amino groups of proteins under hyperglycaemic conditions predominantly trigger oxidative stress and cell dysfunction upon numerous diabetic complications. Mallidis et al also identified these compounds and receptor for AGEs (RAGE) in the male reproductive system, including testes, epididymis and sperm.^10^, ^11^ Karimi et al further confirmed that AGEs played a key role in increasing oxidative stress in the case of reproductive system dysfunction.^12^, ^13^ P38 mitogen‐activated protein kinase (MAPK) is a part of an essential intracellular signalling pathway involved in cell growth, differentiation, development and apoptosis. The phosphorylation of p38 MAPK induced by AGE‐RAGE binding has also been shown to play a central role in various forms of DM‐induced reproductive dysfunction, including testicular damage and erectile dysfunction.^7^, ^9^, ^14^

According to the theory of traditional Chinese medicine (TCM), kidney Yin deficiency is the core pathogenesis of DM‐induced reproductive injury, and consequently, reproductive disorders are commonly treated by tonifying the kidney.^15^ *Cornus officinalis Sieb. et Zucc.* (family: Cornaceae) has often been used in TCM formulations such as Liu‐Wei‐Di‐Huang pills and its derivatives, which can nourish the liver and kidneys, treat impotence, remove internal heat, etc.^16^ *Cornus officinalis* (CO) extract, iridoid glycosides and single compound have been reported to alleviate the damage to diabetic target organs such as the kidneys and testes.^9^, ^17^, ^18^ As a primary bioactive monomer extracted from CO iridoid glycoside,^16^ loganin can inhibit inflammation^19^, ^20^ and protect against DM‐induced nephropathy^21^ and neuropathy.^22^ However, its influence on testicular damage in the context of DM has seldom been referred hitherto. Therefore, we herein, for the first time, used spontaneous type 2 DM (T2DM) model KK‐Ay mice and GC‐2 cells to explore the function and mechanism of loganin in relieving DM‐induced testicular damage and sperm cell apoptosis targeting the AGEs/RAGE/p38MAPK/NF‐κB signalling pathway. The results would provide novel insights into the potential use of loganin to prevent male infertility upon T2DM.

## 2 MATERIALS AND METHODS

### 2.1 Reagents and antibodies

**FIGURE 1:**
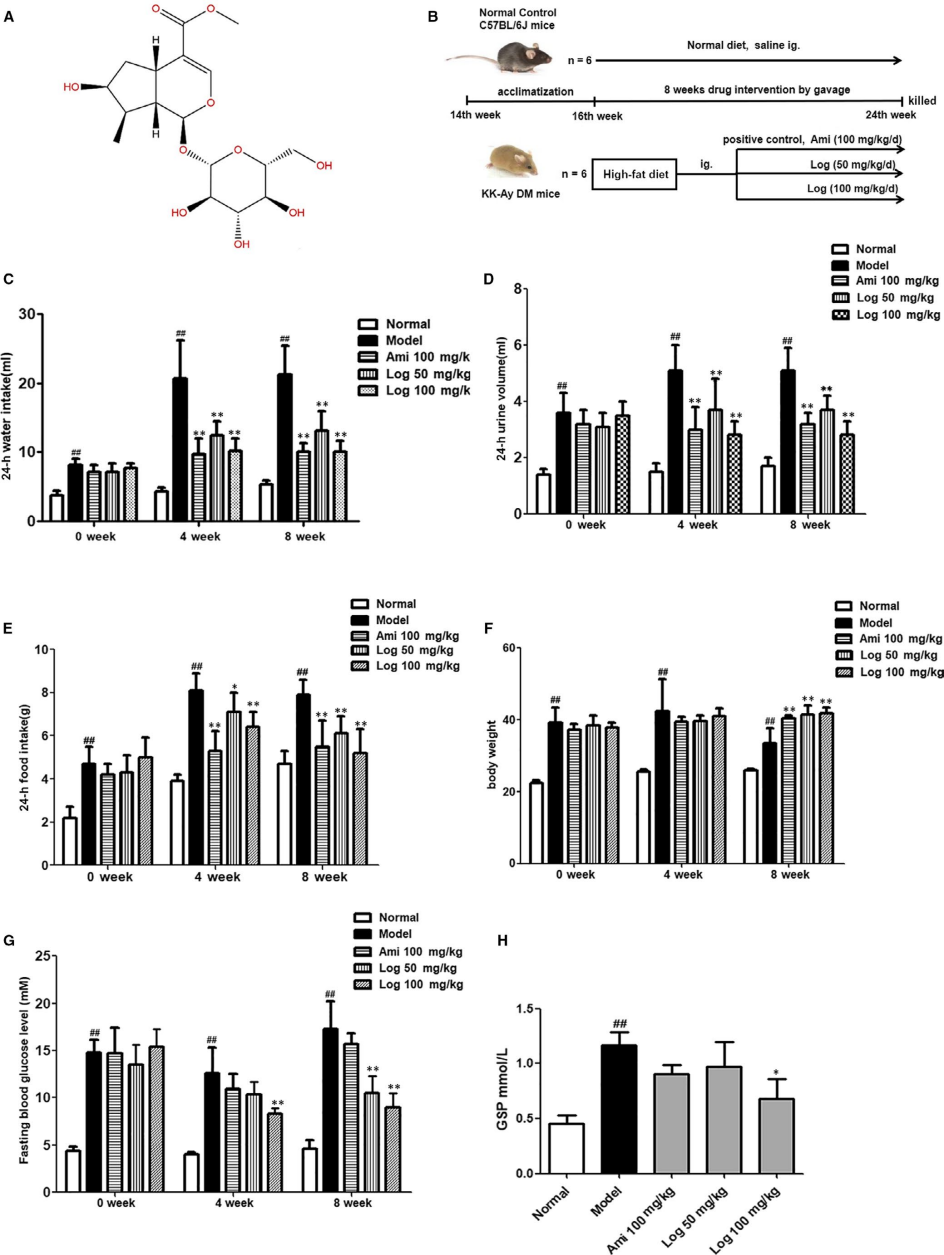
Loganin ameliorated general diabetic symptoms in DM mice. A, Structure of loganin. B, Flow chart of animal study design. C, 24‐h water intake in the fourth and eighth weeks of experiment. D, 24‐h urine volumes in the fourth and eighth weeks. E, 24‐h food consumptions in the fourth and eighth weeks. F, Bodyweights in the fourth and eighth weeks. G, Fasting blood glucose levels in the fourth and eighth weeks. H, Serum GSP level at the end of experiment. Significance: ^##^ *P* < .01 vs normal group; *P < .05, **P < .01 vs model group

Loganin (Figure 1A; 98% purity, batch No. M‐010‐160516) was obtained from Chengdu Herbpurify Co., Ltd. In all experiments, loganin was dissolved in sterile water. Aminoguanidine (98% purity, batch No. 079K1734V) was obtained from Sigma (USA). Antibodies against RAGE (batch No. ab3611), p65 NF‐κB (batch No. ab16502) and Bcl‐2 (batch No. ab196495) were purchased from Abcam, and those against phospho‐p38 MAPK (batch No. 4511S), p38 MAPK (batch No. 9212S), phospho‐p65 NF‐κB (batch No. 3039s) and Bax (batch No. 2772s) were purchased from Cell Signaling Technology. To prepare AGEs, 50 mg/mL bovine serum albumin (BSA) was incubated with 0.5 mol/L D‐glucose at 37°C for 12 weeks in a sterile environment without light. After the formation of AGEs, the solution was dialysed in 10 mmol/L pH 7.4 phosphate‐buffered saline (PBS) to remove unreacted glucose, and AGE content was determined using an ELISA kit.

### 2.2 Animal model establishment

Fourteen‐week‐old male KK‐Ay mice and C57BL/6J mice (Licence Number: 2014‐0004) were purchased from Beijing Hua Fukang Biotechnology Co., Ltd. The flow chart of animal study design is shown in Figure 1B. KK‐Ay mice were fed high‐fat diet (458 kcal/100 g, containing 10% fat), and C57BL/6J mice were fed normal diet. All mice were housed at (25 ± 1)°C and humidity of (55 ± 5)% in a regular 12 h/12 h light/dark cycle to acclimate for two weeks prior to experiments. The animal studies were conducted in accordance with protocols approved by the Animal Ethic Committee of Nanjing University of Chinese Medicine (code: ACU‐13(20161011)). KK‐Ay mice were randomly divided into four groups (n = 6): DM group, aminoguanidine group (100 mg/kg/d) as the positive control, low‐dose loganin group (50 mg/kg/d) and high‐dose loganin group (100 mg/kg/d). All these groups were fed high‐fat diet. Each treatment group was orally given corresponding agent for eight weeks. Six C57BL/6J mice that were fed normal diet and administered with an equal volume of saline were set as a control group. Bodyweight, 24 hours food consumption, 24 hours water intake, 24 hours urine volume, serum insulin level and fasting blood glucose level were measured every four weeks. At the end of experiment, all mice were killed by cervical dislocation, and blood samples were drawn from the orbit. The blood samples were left still at room temperature for 20 minutes and centrifuged at 3000 *g* for 15 minutes, and then the serum was collected. The testes of each animal were collected and weighed.

### 2.3 Cell culture and treatment

GC‐2 cells (a mouse spermatocyte‐derived cell line; Shanghai Meiya Biotechnology Co. Ltd.) were maintained in RM1640 medium (Gibco, Life Technologies Corporation) containing 10% foetal bovine serum at 37ºC with 5% CO_2_. GC‐2 cells were seeded on a collagen‐coated culture plate (Corning Incorporated, Life Sciences) and cultured until the confluent reached 60%. The cells were grouped as follows: control, AGEs (200 mg/L) and loganin (0.1, 1 and 10 μmol/L). Then, the cells were incubated in serum‐free RPMI‐1640 medium for 24 hours to be kept in the G0 phase, and pre‐treated with loganin (0.1, 1 or 10 μmol/L) for 1 hour. Afterwards, AGEs (200 mg/L) were added into the medium for 48 hour of culture to induce cell injury. The AGE group was only stimulated with AGEs, and the control group did not receive any stimulation. To clarify the mechanism, RAGE, p38 MAPK and NF‐κB inhibitors (FPS‐ZM1, SB203580, PDTC) plus loganin were added to the culture medium at the same time 1 hour before stimulation with AGEs (200 mg/L), and the cells were harvested 48 hours after AGEs were added.

### 2.4 Determination of live sperm rate

The live sperm rate was determined according to the method described by Giribabu et al.^23^ The epididymides were homogenized and suspended with PBS. Then, 100 μL of the suspension was mixed with an equal volume of 1% trypan blue in the same medium. Live sperm totally excluded the dye, whereas dead sperm accumulated the dye and exhibited blue heads. Live sperm were analysed under a light microscope with 200× magnification and expressed as a percentage of the total sperm count.

### 2.5 Histological analysis and transmission electron microscopy (TEM)

The testes and kidneys were fixed in 10% formalin solution and then embedded in paraffin. The paraffin blocks were cut into 5‐μm‐thick sections and stained with haematoxylin and eosin (HE). Photographs were taken in a blinded manner from randomly selected fields, and representative images of the sections are shown. Testicular pathology was analysed by pathologists according to the hierarchical arrangement and structure of spermatogenic cells, interstitial cell hyperplasia, interstitial vasodilation, hyperaemia, haemorrhage and inflammatory cell infiltration for semi‐quantitative scoring. Kidney pathology was scored on the basis of renal structural integrity, glomerular mesangial hyperplasia, inflammatory cell infiltration and renal tubular epithelial lesions. A score of 0 indicates no lesions, and a score of 6 was assigned to the most serious lesions. For kidney TEM, the renal cortex was cut into 1 mm × 1 mm chunks and fixed with 2.5% glutaraldehyde solution at 4°C for 24 hours, followed by gradient dehydration and imaging of the glomerulus in random fields and representative podocytes.

### 2.6 Measurement of AGEs in testes

AGE levels in the testes were measured using ELISA kit (Nanjing Yifeixue Biotechnology Co., Ltd.) according to the manufacturer's protocol. Briefly, 96‐well plates were coated with primary antibody and 50 μL of the standard, and control or sample was added into each well of the plate and incubated for 30 min at 37°C. After washing three times, 100 μL of enzyme‐conjugated solution was added and incubated for 30 minutes at 37°C. After another three washes, p‐nitrophenyl phosphate substrate diluted in glycine buffer was added. The absorbance was read at 450 nm using a microplate reader.

### 2.7 Measurement of oxidative stress and testicular markers

The levels of ROS, GSH and the activities of SOD, lactate dehydrogenase (LDH), acid phosphatase (ACP) and gamma‐glutamyl transferase (γ‐GT) in testes and GC‐2 cells were measured using kits purchased from Nanjing Jiancheng Bioengineering Institute (Nanjing, China). Intracellular ROS production was estimated by using 2,7‐dichlorofluorescein diacetate (DCF‐DA) as a probe. Briefly, 100 μL of tissue homogenate or cell suspension was incubated with the assay medium (20 mmol/L Tris‐HCl, 130 mmol/L KCl, 5 mmol/L MgCl_2_, 20 mmol/L NaH_2_PO_4_, 30 mmol/L glucose and 5 μmol/L DCF‐DA) at 37°C for 15 minutes. The formation of DCF was measured at the excitation wavelength of 485 nm and the emission wavelength of 525 nm. GSH can react with DTNB to produce a yellow compound which can be quantified at 405 nm. The level of SOD was detected based on its capacity to inhibit the reduction of nitroblue tetrazolium by superoxide and the absorbance at 560 nm. The LDH activity was measured based on the interconversion between pyruvate and lactate. The activity of γ‐GT was detected by a kinetic method measuring the rate of releasing 5‐amino‐2‐nitrobenzoate from L‐gamma‐glutamyl‐3‐carboxy‐4‐nitroanilide at 405 nm. ACP can decompose disodium phenyl phosphate into free phenol and phosphoric acid. Phenol and 4‐aminoantipyrine can be oxidized by potassium cyanide into red quinone derivatives in alkaline solution. The derivatives were quantified using a microplate reader at 520 nm. The experiments were performed in triplicate.

### 2.8 Evaluate of apoptotic testicular cells in KK‐Ay mice by TUNEL staining

Apoptotic testicular cells were measured using TUNEL assay with a kit purchased from Beyotime Biotechnology Co. Ltd. (batch No. C1086, China). Testicular tissue was fixed in 10% paraformaldehyde, embedded in paraffin and then sectioned into 5 μm thick. Briefly, the sections were deparaffinized and rehydrated in xylene and ethanol, followed by incubation with proteinase K working solution at 37°C for 20 minutes. After rinsing with PBS, the samples were reacted with TUNEL reaction mixture (50 µL) at 37°C for 1 hour and rinsed with PBS to stop the reaction. TUNEL‐positive cells were considered apoptotic and quantitatively analysed with ImageJ software.

### 2.9 Evaluate of GC‐2 cell apoptosis by acridine orange and ethidium bromide (AO/EB) staining and flow cytometry (FCM)

AO/EB dual staining was performed at a 1:1 ratio to evaluate the morphological changes of cells due to apoptosis, and flow cytometry was used to determine the rate of apoptosis. GC‐2 cell culture and treatment were the same as those in subsection 2.3. After incubation, the cells were washed with PBS twice, stained with AO/EB (0.1 mg/mL) and observed under a fluorescence microscope at 200× magnification. In addition, the apoptotic cell rate was determined using an annexin V/PI apoptosis detection kit according to the manufacturer's protocol (Nanjing KeyGen Biotech Co., Ltd.). The cells were incubated with annexin V (conjugated with FITC) and PI in dark for 10 minutes, and their fluorescence was then analysed using a flow cytometer (Beckman Colter). The annexin V‐FITC‐positive cells were considered apoptotic.

### 2.10 Western blotting

Testes were homogenized in RIPA buffer containing protease inhibitor. The protein concentration was measured using a BCA assay kit (Pierce). Protein (30‐50 μg/well) was separated by SDS‐polyacrylamide gel and transferred to a PVDF membrane (Millipore) that was then blocked with 5% BSA in Tris‐buffered saline containing 0.05% Tween 20. Target proteins were detected by primary antibodies against RAGE (Abcam, ab3611), phospho‐p38 MAPK (Cell Signaling, 4511S), p38 MAPK (Cell Signaling, 9212S), Bax (Cell Signaling, 2772s), Bcl‐2 (Abcam, ab196495), p65 NF‐κB (Abcam, ab16502) and phospho‐p65 NF‐κB (Cell Signaling, 3039s), and subsequently by horseradish peroxidase‐conjugated secondary antibodies. Protein bands were visualized using chemiluminescence reagent (Millipore). Equivalent loading was confirmed using an antibody against β‐actin, and the levels of target protein bands were densitometrically determined using ImageJ software. Representative blots were obtained from three independent experiments.

### 2.11 Statistical analysis

Data were collected from repeated experiments and are represented as mean ± standard deviation (SD). One‐way ANOVA was used to determine whether a difference was present and if so, the post hoc Tukey's test was used to analyse the differences between groups using SPSS 19.0 software. *P* < .05 was considered statistically significant.

## 3 RESULTS

### 3.1 Loganin ameliorated general diabetic symptoms of KK‐Ay mice

Bodyweight, 24‐hours food consumption, 24‐hours water intake and 24‐hours urine volume in the fourth and eighth weeks after drug administration were measured. Compared with the normal control group, the model mice had significantly (*P* < .01) increased 24 hours food consumption (1.7‐fold), 24‐hours water intake (fourfold) and 24 hours urine volume (threefold) in the eighth week (Figure 1C‐E). Treatment with positive drug aminoguanidine or loganin ameliorated these symptoms and increased the bodyweight to different extents in comparison to those of the model group (Figure 1C‐F). Moreover, the fasting glucose and glycosylated haemoglobin (GSP) levels in model mice were significantly (*P* < .01) elevated (3.8‐fold and 2.6‐fold, respectively) compared with those of normal mice in the eighth week, which were significantly reduced by loganin (Figure 1G,H).

### 3.2 Loganin mitigated kidney and testicular lesions of KK‐Ay mice

**FIGURE 2:**
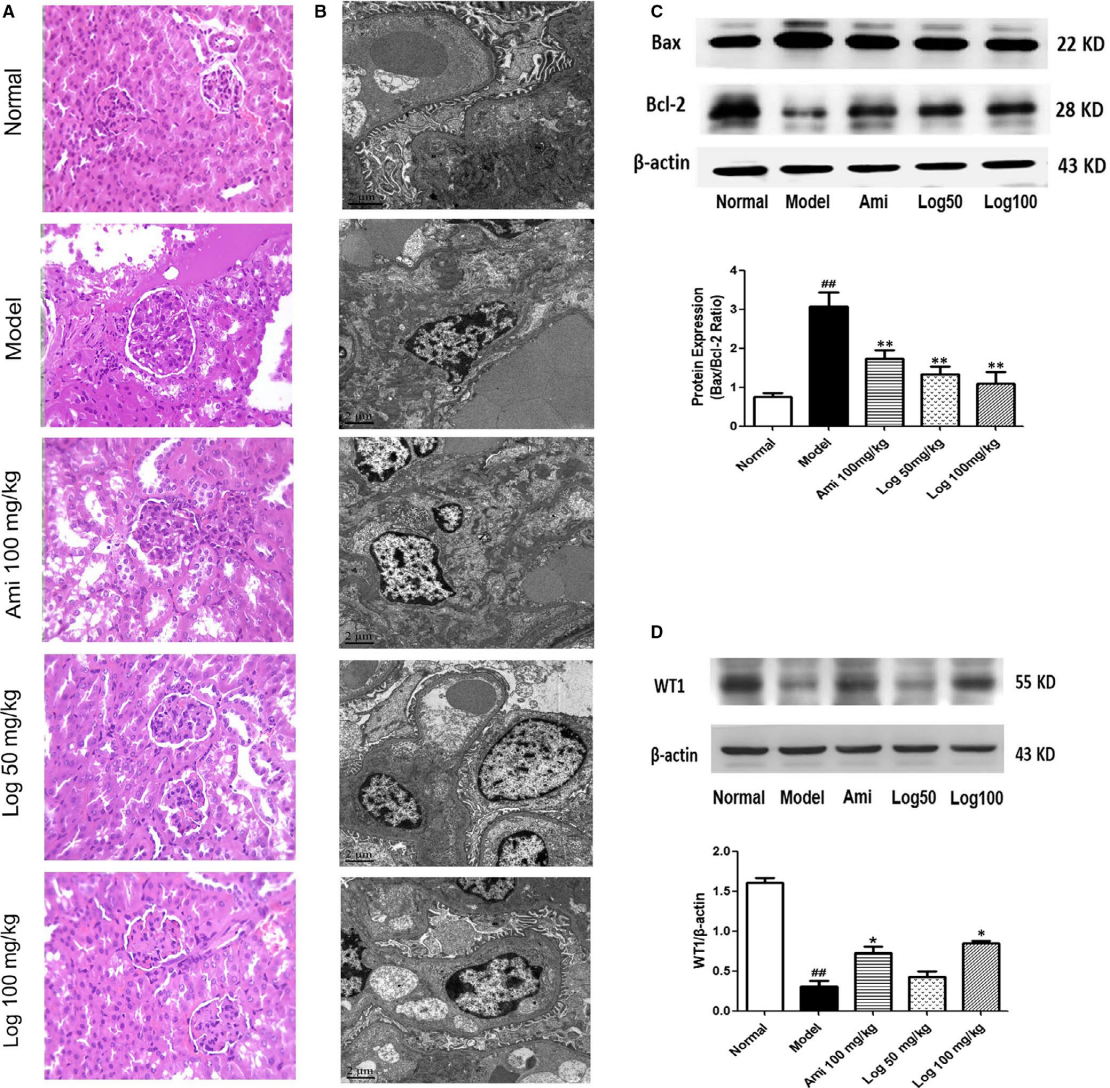
Loganin showed renal protective effects in KK‐Ay mice. A, HE staining of kidney tissues. B, Transmission electron microscopy of glomeruli. C, Western blot analyses of Bax and Bcl‐2 protein expression in kidney cortex. The Bax/Bcl‐2 ratio was calculated and analysed. D, Western blot analyses of WT1 protein expression in kidney cortex. Bars represent the mean ± SD, n = 3. Significance: ^##^ *P* < .01 vs the normal group, *P < .05, **P < .01 vs the model group

**FIGURE 3:**
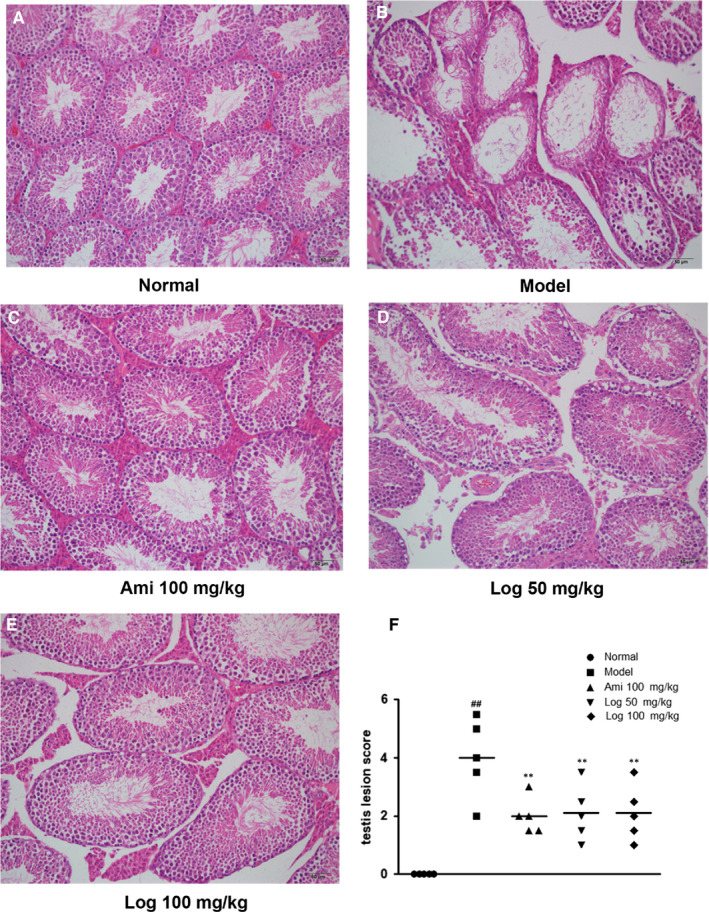
Loganin alleviated DM‐induced histopathological changes in the testes. A‐E, Representative images of HE sections in the testes (200 × magnification). F, Statistical chart of testicular lesion scores vs model group. Significance: ^##^ *P* < .01 vs normal group; **P < .01 vs model group

Since reproduction is closely related to renal function in TCM, we also evaluated the effects of loganin on kidney lesions in KK‐Ay DM mice. HE staining and TEM were used to assess morphological changes, which showed obvious lesions with thickening of the glomerular basement membrane, glomerular hypertrophy and fusion of podocyte foot processes in model mice (Figure 2A,B). In the testis, the control mice exhibited normal testicular structure with intact seminiferous tubules and organized germ cells in concentric layers. Spermatogenic cells at various stages of division (from spermatogonia to spermatid) were observed in neatly arranged seminiferous tubules, with small amounts of interstitial cells in the lumen (Figure 3A). In the model group, the seminiferous tubules were disrupted, accompanied by germ cell degeneration, structural shrinkage and separation from each other (Figure 3B). However, these pathological changes were alleviated to different extents by treatment with positive drug aminoguanidine or loganin for eight weeks (Figures 2A,B and 2, 3). The testicular lesion scores are shown in Figure 3F. Furthermore, the expressions of pro‐apoptotic protein Bax and anti‐apoptotic protein Bcl‐2 in the renal cortex were measured to evaluate apoptosis, and WT1 expression was measured to assess podocyte loss. The Bax/Bcl‐2 ratio of the model group significantly exceeded that of the normal group, which was reversed by using aminoguanidine or loganin (Figure 2C). WT1, a specific marker protein for podocytes, was down‐regulated in the model group in comparison with that of the normal group, which was restored by aminoguanidine or high‐dose loganin treatment (Figure 2D).

**FIGURE 4:**
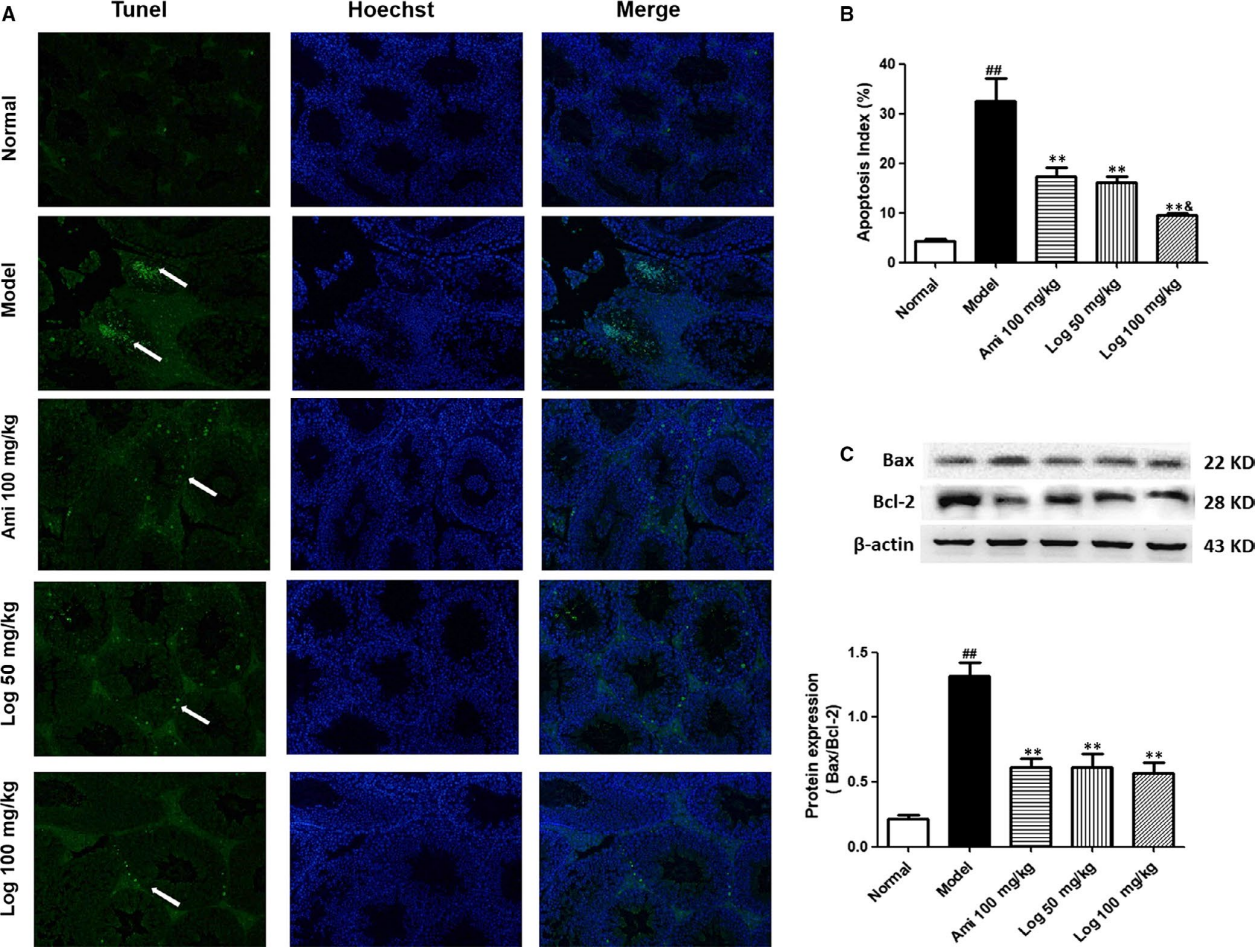
Loganin relieved apoptosis in the testes of KK‐Ay mice detected by TUNEL staining. A, TUNEL‐positive cells (white arrows) in representative images are shown in green (200 × magnification). Cell nucleus was subjected to Hoechst staining. B, Quantification of apoptotic cell percentage using Image J software. C, Western blot analyses of Bax and Bcl‐2 protein expressions in testis homogenate. Bax/Bcl‐2 ratio was calculated and analyzed. Bars represent mean ± SD, n = 3. Significance: ^##^ *P* < 0.01 vs normal group; *P < 0.05, **P < 0.01 vs model group; ^&^ *P* < 0.05 vs aminoguanidine group

### 3.3 Loganin attenuated apoptosis in testes of KK‐Ay mice and AGE‐induced GC‐2 cells

The TUNEL assay revealed numerous apoptotic cells in the testes of the model group, and the apoptosis was mitigated by aminoguanidine or loganin administration (Figure 4A,B). Additionally, the Bax/Bcl‐2 ratio of model mice was significantly (*P* < .01) elevated (Figure 4C) compared with that of the normal control group, which was effectively reversed by aminoguanidine or loganin administration (*P* < .01). Therefore, loganin exerted an anti‐apoptotic effect on DM‐induced testicular damage.

**FIGURE 5:**
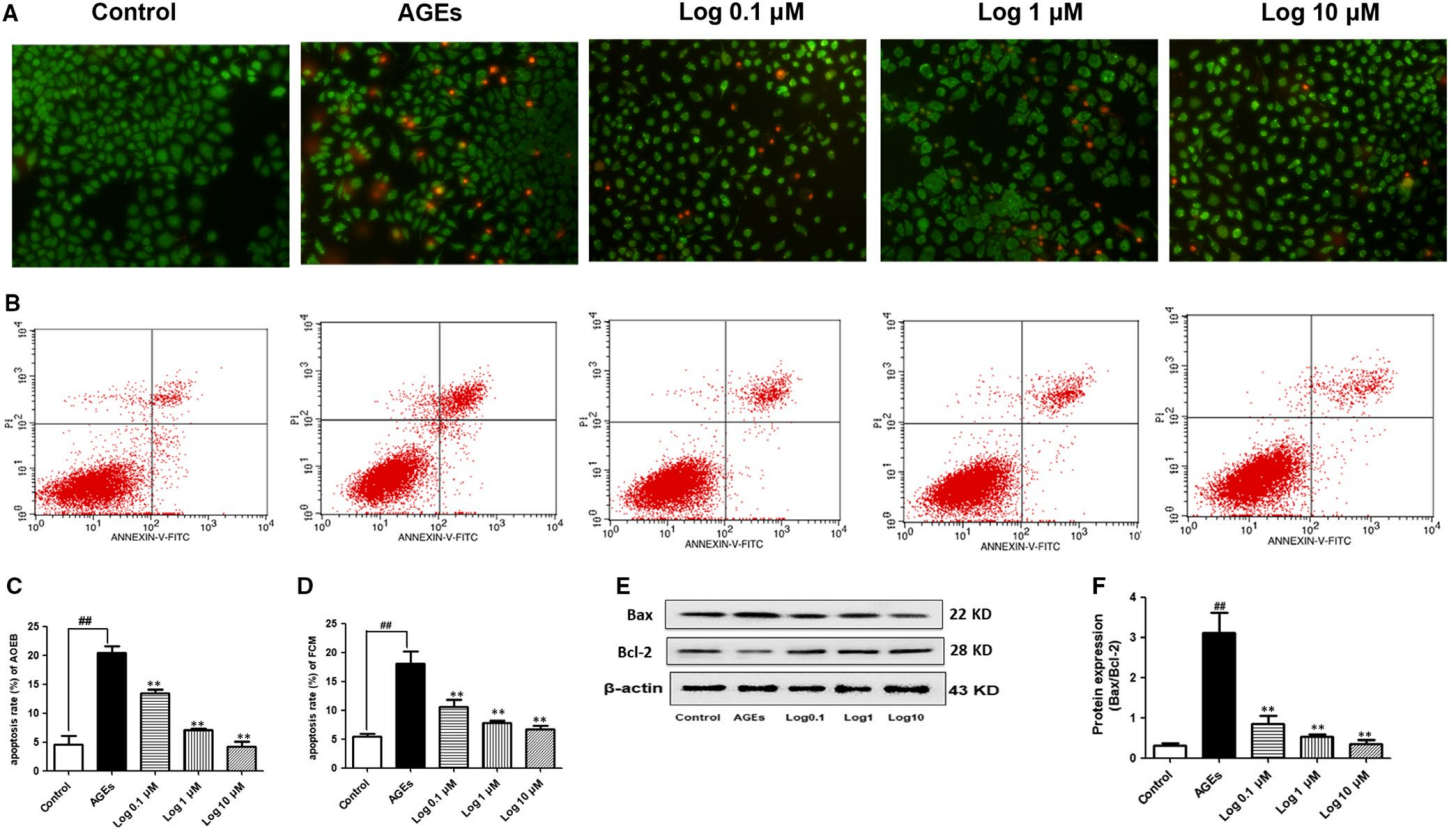
Loganin relieved AGE‐induced GC‐2 cell apoptosis detected by AO/EB staining and FCM. A, AO/EB staining showed that apoptotic cells emitted orange fluorescence and normal cells emitted green fluorescence (200 × magnification). B, FCM of GC‐2 cells treated with loganin. C, Statistical chart of AO/EB staining. D, Statistical chart of FCM. (E and F) Western blot analyses of Bax and Bcl‐2 protein expressions in GC‐2 cells. Bax/Bcl‐2 ratio was calculated and analysed. Bars represent mean ± SD, n = 3. Significance: ^##^ *P* < .01 vs control group; **P < .01 vs AGEs group

AO/EB staining and FCM were performed to evaluate the in vitro protective effects of loganin on GC‐2 cell apoptosis induced with AGEs (200 mg/L) for 48 hours. The apoptosis rate of GC‐2 cells after AGEs treatment was significantly higher than that of the control group. However, pre‐treatment with loganin (0.1, 1 and 10 μmol/L) decreased the apoptosis rate significantly (*P* < .01) in comparison with that of the AGEs group (Figure 5A‐D). Furthermore, the Bax/Bcl‐2 ratio was increased 9.7‐fold after AGE stimulation (*P* < .01), which was effectively reversed by loganin administration (*P* < .01, Figure 5E,F). Thus, loganin exerted an anti‐apoptotic effect on GC‐2 cells stimulated with AGEs.

**FIGURE 6:**
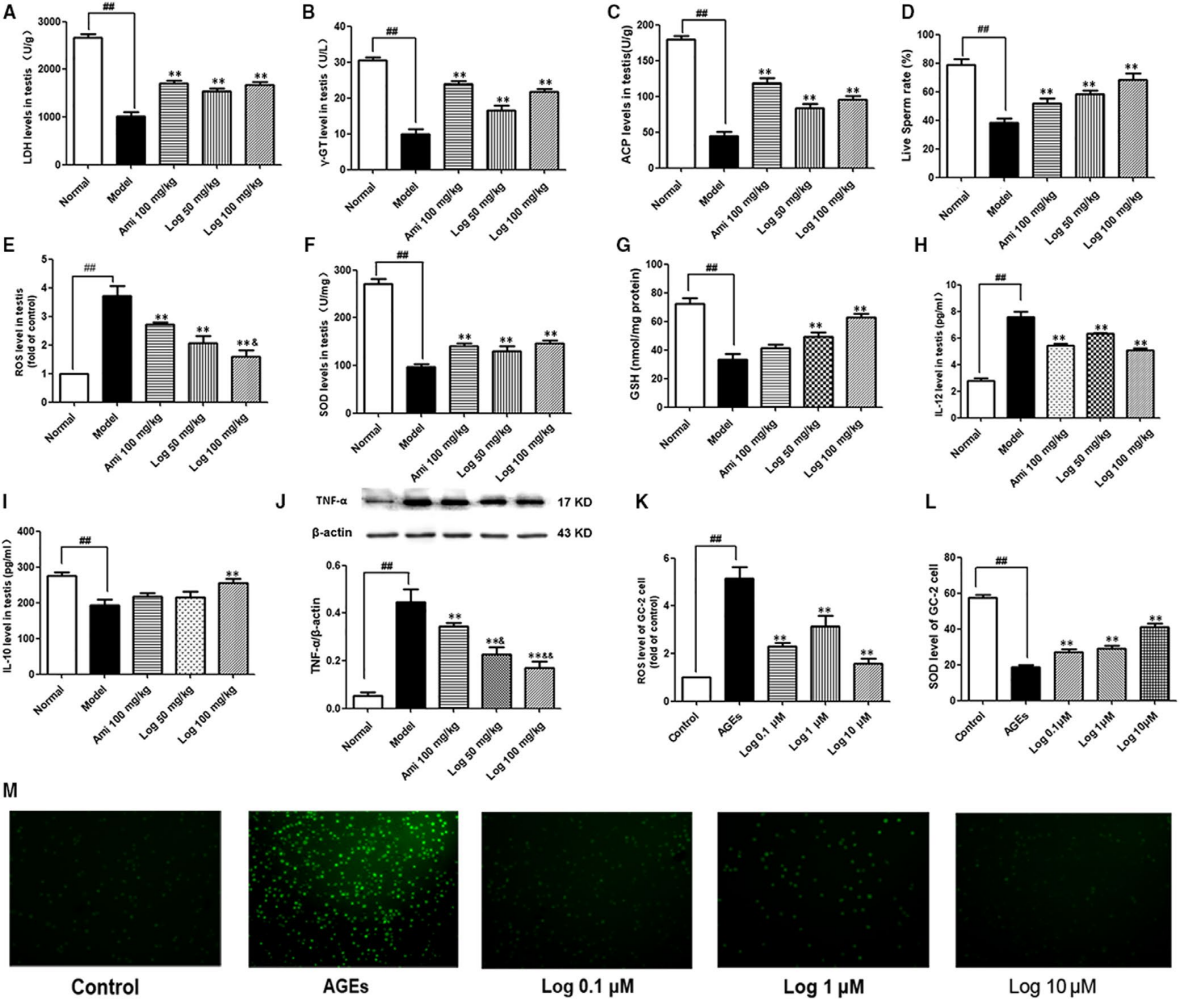
Loganin improved oxidative stress, inflammatory response and down‐regulated testis‐specific enzymes. A, LDH level in testis homogenate. B, γ‐GT level in testis homogenate. C, ACP level in testis homogenate. D, Live sperm rate. E, ROS level in testis homogenate. F, SOD level in testis homogenate. G, GSH level in testis homogenate. H, IL‐12 level in testis homogenate. I, IL‐10 level in testis homogenate. J, TNF‐α protein expression in the testis. (K and M) ROS fluorescence intensity in GC‐2 cells and statistical chart. L, SOD level in GC‐2 cells. Bars represent mean ± SD, n = 3. Significance: ^##^ *P* < .01 vs normal or control group; **P < .01 vs model or AGE group; ^&^ *P* < .05, ^&&^ *P* < .01 vs aminoguanidine group

### 3.4 Loganin improved oxidative stress and inflammatory responses and down‐regulated testis‐specific enzymes

Testis‐specific marker enzymes LDH, ACP and γ‐GT significantly decreased (*P* < .01) in model mice (Figure 6A‐C) compared with those in normal control mice. Aminoguanidine and loganin treatment up‐regulated their levels to various extents in comparison to those of the model group (Figure 6A‐C). Furthermore, the live sperm rate of the model group decreased by 51.2% (*P* < .01) compared with that of normal mice (Figure 6D), which was raised by treatment with aminoguanidine or loganin. Besides, the model mice and AGE‐induced cells had significantly decreased SOD activity as well as increased ROS level (*P* < .01) and fluorescence intensity (Figure 6E‐G,K‐M), and the GSH level in the testes of model mice was also down‐regulated compared with that of the normal group. Compared with the model group, the mice or cells treated with loganin had significantly restored SOD activity and GSH level together with reduced ROS level (Figure 6E‐G,K‐M). To evaluate the anti‐inflammatory effect of loganin, the protein expression levels of pro‐inflammatory cytokines IL‐12 and TNF‐α together with anti‐inflammatory cytokine IL‐10 in testis homogenate were measured by ELISA. Compared with the normal group, the levels of IL‐12 and TNF‐α significantly increased, and that of IL‐10 markedly decreased, which were reversed by aminoguanidine or loganin. Particularly, loganin better reduced TNF‐α expression (Figure 6H‐J).

**FIGURE 7:**
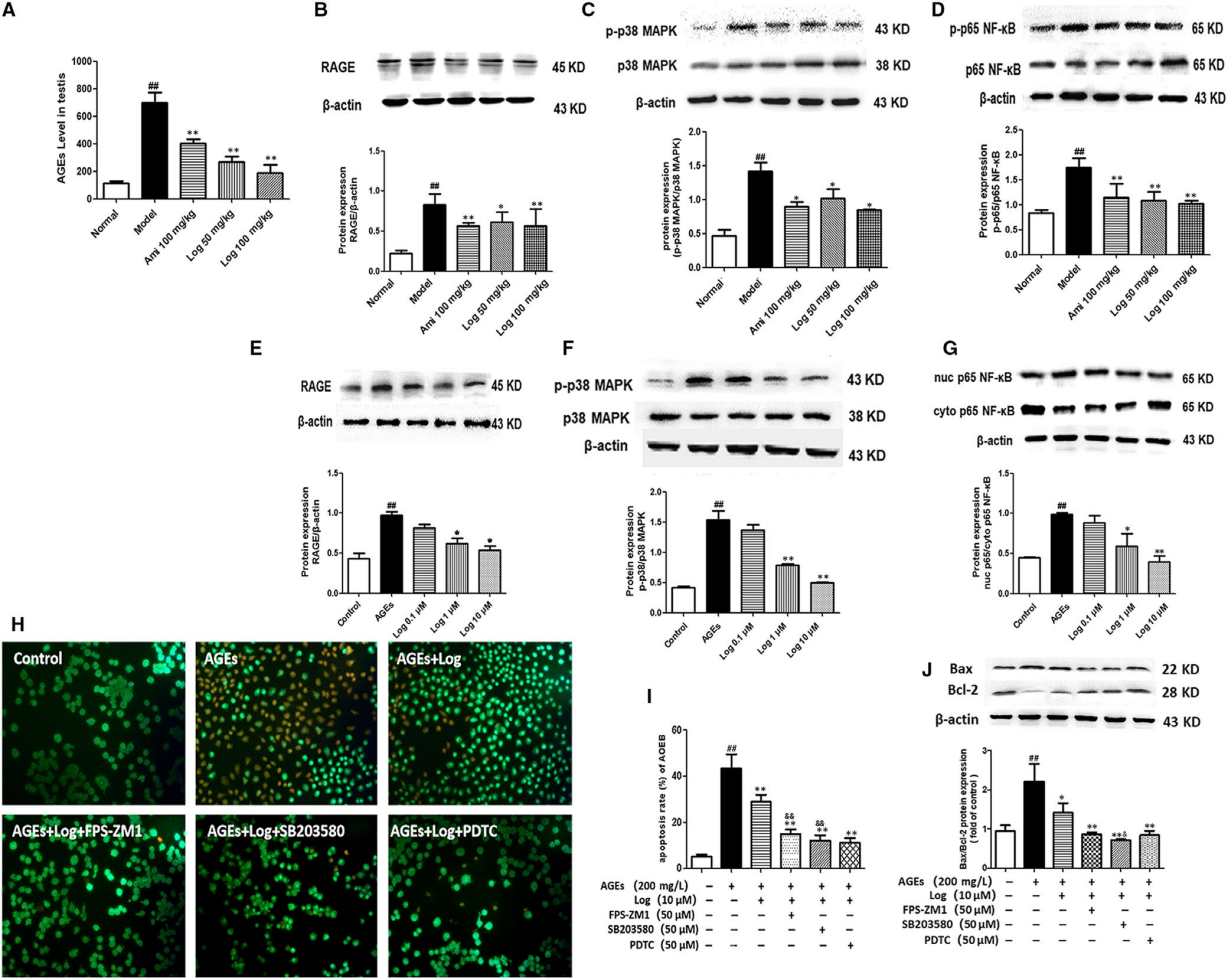
Loganin inhibited the AGEs‐RAGE/p38 MAPK/p65 NF‐κB pathway in DM mice and GC‐2 cells. A, AGE level in the testis. B, Western blot analysis of RAGE protein expression in the testis. C, Western blot analysis of p38 MAPK phosphorylation in the testis and statistic chart of p‐p38MAPK/p38MAPK ratio. D, Western blot analysis of p65 NF‐κB phosphorylation in the testis and statistical chart of p‐p65 NF‐κB/p65 NF‐κB ratio. E, Western blot analysis of RAGE protein expression in GC‐2 cells. F, Western blot analysis of p38 MAPK phosphorylation in GC‐2 cells and statistical chart of p‐p38MAPK/p38MAPK ratio. G, Western blot analysis of nuclear p65 NF‐κB translocation in GC‐2 cells and statistical chart of nuclear/cytoplasmic p65 NF‐κB ratio. (H and I) GC‐2 cells were pre‐incubated with loganin only or in combination with inhibitors of RAGE, p38 MAPK and NF‐κB (FPS‐ZM1, SB203580, PDTC) for 1 h, and then stimulated with AGEs (200 mg/L) for 48 h before AO/EB staining. J, Western blot analyses of Bax and Bcl‐2 expressions in GC‐2 cells pre‐incubated with loganin alone or plus inhibitor of either FPS‐ZM1 or SB203580 or PDTC. Bax/Bcl‐2 ratio was calculated and analysed. Bars represent mean ± SD, n = 3. Significance: ^##^ *P* < .01 vs normal group; *P < .05, **P < .01 vs model group; ^&^ *P* < .05, ^&&^ *P* < .01 vs aminoguanidine group

### 3.5 Loganin inhibited the activated AGEs‐RAGE/p38 MAPK/p65 NF‐κB pathway in DM mice and GC‐2 cells

To further explore the mechanism underlying the protective effects of loganin, we measured the levels of AGEs and RAGE in the testes. The phosphorylation of p38 MAPK and p65 NF‐κB, one of the downstream pathways for RAGE closely related to the induction of inflammation and apoptosis, was also tested. The model group had higher levels of AGEs in the testes than those of normal controls, but aminoguanidine or loganin attenuated the deposition of AGEs to various degrees (Figure 7A). Western blot exhibited that compared with normal mice, RAGE expression was elevated in model mice (*P* < .01), but significantly down‐regulated by aminoguanidine or loganin (Figure 7B). Additionally, the phosphorylation of p38 MAPK and p65 NF‐κB was enhanced in model mice compared to that of the normal group, which was significantly attenuated by treatment with aminoguanidine or loganin for eight weeks (Figure 7C,D).

To further explore the mechanism for the protective effects of loganin on DM‐induced testicular damage based on the AGEs‐RAGE pathway, GC‐2 cells were further treated with AGEs (200 mg/L) for 48 hours to induce germ cell apoptosis. Compared with the control group, the AGE group had markedly up‐regulated RAGE protein expression and phosphorylation of p38 MAPK (*P* < .01; Figure 7E,F), as well as augmented expression and nuclear translocation of p65 NF‐κB and nuclear/cytoplasmic ratio (*P* < .01; Figure 7G). The cells exposed to pre‐treatment with loganin (0.1, 1 or 10 μmol/L), especially that at 10 μmol/L, had significantly decreased RAGE expression and phosphorylation of p38 MAPK compared with those of the AGE group (Figure 7E,F). Additionally, the nuclear translocation of p65 NF‐κB was also significantly attenuated in the 10 μmol/L loganin group (*P* < .01, Figure 7G).

To verify the role of the AGEs‐RAGE/p38 MAPK/p65 NF‐κB signalling pathway in the protective effects of loganin on DM‐induced testicular damage, GC‐2 cells were pre‐treated with loganin (10 µmol/L) plus specific inhibitors, including RAGE blocking agent FPS‐ZM1, p38 MAPK inhibitor SB203580 or p65 NF‐κB inhibitor PDTC, for 1 hour. Then, the cells were stimulated with AGEs (200 mg/L) for 48 hours before AO/EB staining. Combining loganin with FPS‐ZM1, SB203580 or PDTC exerted a similarly stronger anti‐apoptotic effect than that of loganin alone (*P* < .01, Figure 7H,I). Furthermore, the combination of loganin with inhibitors significantly decreased the Bax/Bcl‐2 ratio compared with that of the group administered with loganin only (Figure 7J). Collectively, the anti‐apoptotic effect of loganin was associated with suppression of the AGEs‐RAGE/p38 MAPK/p65 NF‐κB signalling pathway.

## 4 DISCUSSION

Diabetes mellitus is a chronic metabolic disease causing multiple organ and system dysfunction, including kidney failure, retinopathy and so on. With increasingly younger age of onset, the effect of DM on the male reproductive system has gradually aroused concern. DM may compromise male reproduction function at multiple levels, such as endocrine secretion, spermatogenesis, penile erection and ejaculation.^5^ The effects of DM on sperm count, motility and morphology have been widely studied,^24^, ^25^ but the results are inconsistent and the treatment or mechanism of DM‐induced reproductive damage remains elusive.

In this study, we selected spontaneous DM model KK‐Ay mice which had marked obesity, glucose intolerance, insulin resistance and hypertension, all being the characteristics of T2DM phenotype.^26^ Zhou et al also reported serious kidney injury in KK‐Ay mice, manifested as albuminuria, glomerular hypertrophy and mesangial matrix accumulation.^27^ Our study showed that 14‐week‐old male KK‐Ay mice which received high‐fat diet exhibited typical diabetic symptoms, that is polyuria, polydipsia, polyphagia and increase in blood glucose level, accompanied by damage to the morphology and function of the reproductive system. HE staining of the testes in DM mice showed apparent pathological changes, including seminiferous tubule sloughing or atrophy and germ cell degeneration, together with the shrinkage and separation of seminiferous tubule structures. Moreover, the sperm count and live sperm rate of DM mice significantly decreased. Compared with the control group, the DM group had higher apoptotic rate of spermatogenic cells, down‐regulated expression of anti‐apoptotic protein Bcl‐2, up‐regulated expression of pro‐apoptotic protein Bax, as well as significantly raised Bax/Bcl‐2 ratio. Taken together, DM can cause male reproductive damage.

Loganin is an iridoid glycoside extracted from CO which has diverse cell protective functions. For example, loganin exerts beneficial anti‐inflammatory effects by down‐regulating TNF‐α, MCP‐1 and IL‐6 expressions and suppressing activation of the NF‐κB signalling pathway.^19^, ^20^ Furthermore, Hwang et al found that loganin effectively recovered the learning and memory damage induced by scopolamine.^28^ Our group has previously reported that loganin relieved the diabetic nephropathy symptoms of T2DM mice, which was associated with the inhibition of AGE‐RAGE interaction.^21^ Since kidney function is closely related to reproduction in the TCM theory,^15^ we herein used a KK‐Ay mouse model of T2DM to assess the protective effect of the kidney‐tonifying drug loganin on reproductive disturbances.

We found that loganin alleviated the general diabetic symptoms of KK‐Ay DM mice, such as polyuria, polydipsia, polyphagia and elevation of fasting blood glucose level, attenuated the pathological lesion of kidney and decreased the number of podocytes. The reproductive damage and pathohistological alterations in these mice were also mitigated by loganin, as evidenced by the restored live sperm rate, testicular oxidative stress index, inflammatory indices, tissue‐specific enzymes and apoptosis. Consistently, Liu et al reported that the decreased LDH, ACP and γ‐GT activities in the testes of DM rats were reversed by the total saponins from CO.^17^ Park et al demonstrated that the administration of loganin isolated from CO had a protective effect on hepatic oxidative stress upon T2DM by regulating the expressions of proteins related to oxidative stress, inflammation and apoptosis.^29^ Loganin can also effectively resist apoptosis by lowering TNF‐α‐induced expressions of caspase‐3, cleaved caspase‐8 and cleaved caspase‐7 in SW10 cells.^30^ In this study, loganin significantly recovered the decreased activities of testis‐specific enzymes LDH, ACP and γ‐GT in the testes of DM mice. As the main trigger of diabetic complications, ROS increased markedly in the testes of KK‐Ay DM mice, and the SOD activity and GSH level decreased. In contrast, oxidative stress was significantly attenuated by loganin administration. Excessive apoptosis can cause damage to sperm production and semen quality, resulting in oligozoospermia and asthenospermia.^31^ The apoptotic rate of testicular cells in DM mice exceeded that of the normal group, which was significantly reduced by loganin intervention. Meanwhile, the Bax/Bcl‐2 ratio, which increased markedly in DM mice compared with that in the normal control group, decreased after loganin treatment, further verifying the anti‐apoptotic effect of this drug. Moreover, loganin combated inflammation by down‐regulating pro‐inflammatory cytokines IL‐12 and TNF‐α and up‐regulating anti‐inflammatory cytokine IL‐10 in the testes of DM mice. As suggested by HE staining, loganin managed to protect against reproductive damage. In short, loganin may be a promising candidate for the treatment of DM‐induced reproductive damage, but the detailed mechanism of this protective action has not yet been clarified.

One of the important alterations in the case of prolonged hyperglycaemia is the generation and accumulation of AGEs which form through a series of reactions from Schiff bases to stable irreversible end products. Both AGEs and their receptors dominantly participate in the pathogenesis of DM and its complications.^32^ The most studied AGE receptor is RAGE which is expressed on several types of cells such as macrophages,^33^, ^34^ T lymphocytes,^35^ endothelial cells,^36^ podocytes^37^, ^38^ and smooth muscle cells.^39^ The AGEs‐RAGE interaction triggers key cell signalling pathways such as p21ras, MAPK, NADPH and NF‐κB, leading to the activation of proinflammatory responses.^40^ Besides, AGEs‐RAGE and its downstream pathways are essentially involved in DM‐induced reproductive disorders. The binding of AGEs to RAGE induces oxidative stress and then enhances lipid peroxidation in the semen of diabetic men, thereby playing a central role in the pathogenesis of DM‐induced reproductive damage.^41^ Herein, treating DM mice with loganin for eight weeks significantly attenuated the elevation of AGE and RAGE expression levels in the testes and enhanced the phosphorylation of p38 MAPK and p65 NF‐κB. As an AGE inhibitor, aminoguanidine also showed protective effects on reproductive functions by combating oxidative stress and apoptosis. In addition, aminoguanidine significantly inhibited the AGEs‐RAGE/p38MAPK/NF‐κB pathway. Likewise, Orman et al found that activation of the AGEs‐RAGE/p38MAPK/NF‐κB pathway aggravated reproductive damage.^42^ To elucidate the role of AGEs‐RAGE in diabetic reproductive disturbances, a mouse spermatocyte‐derived cell line GC‐2 was treated with AGEs to induce damage in vitro. The apoptosis rate and oxidative stress of AGE‐induced GC‐2 cells were increased compared with those of the blank control group, which were ameliorated by loganin intervention. In the AGE‐induced GC‐2 cell damage model, the level of RAGE, the phosphorylation of p38 MAPK and the nuclear translocation of p65 NF‐κB were elevated in the AGE group, indicating that the RAGE/p38MAPK/NF‐κB pathway was activated after AGE treatment. However, pre‐treatment with loganin before AGE stimulation blocked this activation and relieved AGE‐induced cell damage. Notably, the administration of loganin along with RAGE blocking agent FPS‐ZM1, p38 MAPK inhibitor SB203580 or p65 NF‐κB inhibitor PDTC better suppressed apoptosis than loganin alone. Hence, activation of the AGEs/RAGE/p38MAPK/NF‐κB signalling pathway indeed played a key role in the progression of DM‐induced testicular damage in mice, which may be inhibited by loganin to confer protection against this damage.

In summary, loganin significantly mitigated DM‐induced reproductive damage by improving diabetic metabolic parameters, protecting testicular structure and function, as well as attenuating oxidative stress, inflammation and apoptosis in the testes. Loganin also reduced the level of AGEs, and inhibited the protein expression of RAGE and its downstream p38MAPK/NF‐κB signalling pathway, which may be related to DM‐induced oxidative stress, inflammation and apoptosis in the testes. Further in‐depth studies are still needed to clarify the mechanism by which loganin protects against DM‐induced reproductive disorders.

## CONFLICT OF INTEREST

The authors all declare that they have no competing interests.

## AUTHOR CONTRIBUTIONS

Participated in research design: HX, JHS, YC. Conducted experiments: YC, NJ, YZ, JC, YF and MJ. Contributed new reagents or analytic tools: HW. Performed data analysis: LL and QD. Wrote or contributed to the writing of the manuscript: YC, and HX.
